# The Study of HLA-G Gene and Protein Expression in Patients with Recurrent Miscarriage

**DOI:** 10.15171/apb.2019.009

**Published:** 2019-02-21

**Authors:** Elnaz Mosaferi, Nazila Alizadeh Gharamaleki, Laya Farzadi, Jafar Majidi, Zohreh Babaloo, Tohid kazemi, Mehrnoosh Ramezani, Meraj Tabatabaei, Hamid Ahmadi, Leili Aghebati maleki, Behzad Baradaran

**Affiliations:** ^1^Immunology Research Center, Tabriz University of Medical Sciences, Tabriz, Iran.; ^2^Women Reproductive Health Research Center, Tabriz University of Medical Sciences, Tabriz, Iran.; ^3^Department of Biochemistry, school of Medicine, Ardabil University of Medical Science, Ardabil Iran.; ^4^Department of Immunology, School of Medicine, Shahid Beheshti University of Medical Sciences, Tehran, Iran.; ^5^Department of Anatomy, School of Medicine, Tehran University of Medical Sciences, Tehran, Iran.

**Keywords:** HLA-G, placenta, Real-Time PCR, Miscarriage

## Abstract

***Purpose:*** Although it has been frequently confirmed that HLA-G plays an important role in the reproduction and pregnancy, the pattern of HLA-G gene and its protein expression are rarely addressed in studies. Therefore we conducted this study in regard to evaluate the HLA-G gene and its protein expression in the women’s placenta with recurrent miscarriage.

***Methods:*** Placental samples were obtained from the women who were admitted for delivery or abortion in Al Zahra and Taleghani hospitals, Tabriz, Iran. HLA-G gene expression was determined by real-time polymerase chain reaction (PCR) and HLA-G protein expression was assessed by western blotting and immunofluorescence staining in the tissue samples.

***Results:*** The results showed a significant decrease in the expression of gene and proteins of HLA-G in the women with recurrent miscarriage compared to the control placental tissues.

***Conclusion:*** According to the obtained results, it was concluded that the decrement of HLA-G gene and protein expressions are associated with recurrent miscarriage. Since there are conflicting results from other studies, it is suggested to conduct a more comprehensive similar study with greater sample size.

## Introduction

Recurrent miscarriage (RM) refers to ≥3 consecutive fetus losses in first-trimester or ≥2 in the second-trimester that about 1%-2% of all women confronted with it.^1^ About 50% of RM cases are associated with the known causes including thrombophilic disorders (40%), uterus malformations (19.3%), humoral dysfunction (17%-20%), parental chromosome disorders (4%) and the dysfunction of maternal immune system (3%-5%). Although in approximately 50% of RM cases, the etiology still remains unknown and are classified as idiopathic.^2^ However, most idiopathic RM cases are considered as a result of maternal immunological graft rejection response against fetus and placenta.^3^ The local compatibility of maternal immune system with semi allograft fetus leads to the successful mutual adaptation between mother and a fetus, which expresses the maternal and paternal gens in fifty-fifty manner.^4^ Regarding to the achievement of such compatibility, the cytotoxic response of acquired immunity is modulated or ever inactivated and regulatory responses are enhanced.^5,6^ In contrast, intrinsic immune responses remain intact in order to provide the mother’s immune system against infections and interact with embryonic tissues to form placenta and successful pregnancies.^7-10^

It is suggested that these regulatory mechanisms are devastated in most cases of idiopathic RM cases, and subsequently, the maternal immune system responses to the fetus’s antigens. Immunosuppression of leukocytes in maternofetal interface is one of the main mechanisms for modulation of maternal immune system against semi allograft fetus. In the first trimester, 10%-15% of total placenta cells are compromised by leukocytes (killer cells and T cells) that are accounting for about 70% and 10% of this population, respectively.^11^ The uterine NK cells are distinct CD56^+^CD16- NK cells with suppressor receptors, which bind to HLA-G.^12^

Growing evidence suggests that placental-expressed nonclassical class I MHC molecules such as HLA-E, F and most importantly HLA-G may play crucial roles in modulation of maternal immune system.^13-15^

Different studies suggested that HLA-G plays an important role in different stages of reproduction and even before fertilization. HLA-G is expressed on extra villous trophoblastic and amniotic epithelial cells, which has an essential role in a successful pregnancy.^14,16,17^ It has been shown that sHLA-G levels in women’s serum during pregnancy are 2-5 folds more than non-pregnant ones.^18^

The association of HLA-G with pregnancy complications such as preeclampsia and RM has been proposed in different publications. Also, it is reported that various polymorphisms and alteration in transcription of HLA-G gene is related to the increment of risk of these disorders.^19-21^ Also, some studies showed that increasing of uterus NK cells and high expression of HLA-G accompanied with soluble HLA-G is associated with the higher rate of successful pregnancy and disruption of existing balance leads to severe problems in reproduction and pregnancy.^22-24^

Although many reports verified the role of HLA-G in the human reproduction and pregnancy, some studies have been conducted on the pattern of HLA-G genes expression. Therefore, the present study was aimed to evaluate the HL-G genes expression in women’s placenta with RM in comparison with those experienced successful pregnancies.

## Materials and Methods

### Preparation of maternal placental tissue samples

Maternal placental samples were obtained from volunteers that admitted for delivery or abortion in Al-Zahra and Taleghani hospitals, Tabriz–Iran. Twenty-one placental tissue samples of the RM patients (2 or 3 times or more, continuously and under twenty weeks) and 23 placental tissue samples of the normal pregnancies from mothers without any abortion history (as control) were obtained. Following bacterial, viral and karyotype assessments on the aborted samples, there were no sign of pathogenicity and genetic abnormalities, so that there were not any justifying reasons for the abortion. Table 1 presents demographic details of the test group.

**Table 1:** Demographic details of the test group

**Mother's age**	**<30 Years Old**	**>30 Years Old**
	**84%**	**16%**
Gestational age	First trimester	Second trimester
	66%	34%
Frequent RM	Twice	>2
	43%	57%
Duration between 2 RM	< 2 years	> 2 years
	81%	19%

Immediately after sampling, the placenta were put in normal saline at 4°C and far from light. Then, they were transferred to a laboratory, after washing in normal saline.

### RNA extraction and cDNA synthesis

RNA extraction was performed by using RNX Plus (Cinnagen, Iran) according to the manufacturer’s instruction under RNAase free condition. The concentration and purity of the extracted RNA was detected (A260/A280) by Biophotometer (Eppendorf, Germany) and its quality was approved by electrophoresis. cDNA was synthesized by Revertaid^TM^ first strand cDNA synthesis (Fermentas, USA) according to the manufacturer’s instruction in order to detect the mRNAs of HLA-G and β- Globin, and were stored at -20°C.

### Gene expression

The specific primers for HLA-G were designed by using Fast PCR software (version 6) and their specificity for underlined genes were proven by online BLAST and RT Primer database, then synthesized (Bioneer, South Korea). The sequence of the primers is shown in Table 2.

**Table 2:** The sequence of primers for HLA-G and β-Globin genes

**Genes**	**Sequence of primer**
β-Globin	**F:** ACA CAA CTG TGT TCA CTA GC
	**R:** CAA CTT CAT CCA CGT TCA CC
HLA-G	**F:** TGG AGC AGG AGG GGC CGG AG
	**R:** CCG CGC AGG GTC TGC AGG TT

In order to evaluate the HLA-G and β-Globin gens expression, real-time polymerase chain reaction* (*PCR*)* was conducted. The relative quantitation of Real-Time PCR was performed by measurement of fluorescent radiation that was resulted in cyber green dye binding through Corbett-rotor Gene-600 (Corbett, Life Science, Australia). Total volume of the PCR reaction was 20 µL containing 10 µL of SYBR Green Master Mix (Applied Biosystems, USA), 0.5 µL of the primers mixture, 2 µL of cDNA and 7.5 µL of DEPC water.

The PCR cycle conditions were 10 minutes at 95°C for early denaturation, 20 seconds at 95°C, 20 seconds at 65°C and 20 seconds at 72°C for 45 cycles. Finally, before data analyses, the melting curves of each reaction was assessed in order to prove the accuracy of the related gene peak. Crude data were extracted as Ct and analyzed using RESET (M.Pfaffl -Technical University Munich) and Rotor-Gene Q Series Software (Australia). To normalize the expression of HLA-G gene, β-Globin gene was used. So that in each sample, the ΔCt values for HLA-G gene was calculated as differentiating Ct values for HLA-G gene from that of β-Globin as reference gene.

### Immunoblotting analysis

HLA-G protein expression in the tissue samples was assessed by using Western blot and immunofluorescence techniques. About 150 mg of the placental tissue was mixed with 450 µL of lysis buffer (150 mM Nacl, 50 mM HCL pH = 7.4, 1 mM EDTA, Triton X-100 1%, Sodium deoxycholic acid 1%, SDS and about 0.1% of proteinase inhibitor) in a grinder and digested mechanically at 4°C, then centrifuged by 13000 RPM at 4°C for 10 minutes. The concentration of the obtained proteins was detected by using BCA Protein Assay kit (Pierce, USA). Equal amount (20 μg) of the total proteins from each tissue samples were loaded on each well and separated on 4% SDS polyacrylamide gels via a mingle apparatus (Bio-Rad Laboratories).

The obtained proteins were then transferred to polyvinyl denied fluoride (PVDF) membranes (Millipore; Billerica, MA).

Following that, the membranes were blocked with 5% skim milk in PBS by incubation overnight at 4°C. Then PVDF membrane was incubated overnight at 4°C with the appropriate primary antibodies (anti-HLA-G and β-Actin; Abcam, Cambridge, MA, UK) followed by incubation with the appropriate HRP-conjugated secondary antibody (1:1000 dilution; Abcam) for 1.5 hours at room temperature. After washing the membranes, protein bands were visualized using enhanced chemiluminescence kit (GE healthcare, UK). In order to approve the equality of the loaded proteins among tests and control groups, the immunoblotting of β-Actin protein was used.

### Immunofluorescence staining

The placental tissue specimens were cut into sections with 5-20 µm in thickness, then they were fixed using acetone for 20 minutes at -20°C. The slides were kept for 10 minutes at room temperature. Then, in order to block unspecific binding sites, they were incubated with blocker buffer (5% of sheep serum and 2.5% of BSA in PBS) for 1 h at room temperature. The slides were incubated with anti-human HLA-G antibody for 12 hours at 4°C, and then followed with secondary antibody conjugated to FITC for 1 hours at room temperature. To eliminate non- specific binding of secondary antibody, the negative control (not incubated with anti HLA-G antibody) was used. Finally, the slides were analyzed by using fluorescent microscope (Nikon, Japan).

### Statistical analysis

Statistical analyses were performed using SPSS, PC Statistics (version 19.0; SPSS Inc., Chicago, IL, USA). Paired *t* test was applied to compare the results of studies. *P* values <0.05 were reported to be statistically significant.

## Results and Discussion

**Figure 1:**
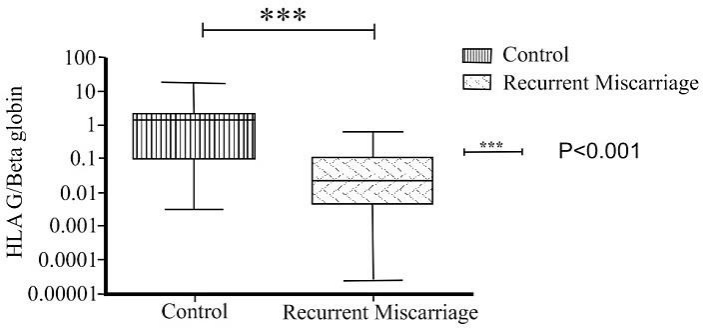
The graph related to comparison of HLA-G expression between test and control groups. According to the results of Real-Time PCR, the HLA-G gene expression was decreased significantly in the test group.

The rate of HLA-G expression was assessed in the 21 women’s placental samples which experienced ≥3 RM, and in the 23 women with successful pregnancies (without any miscarriage) as control group. According to the results of real-time PCR, the expression of HLA-G gene was significantly decreased in the test group (*P *˂ 0.001(. The results are presented in Figure 1.

**Figure 2:**
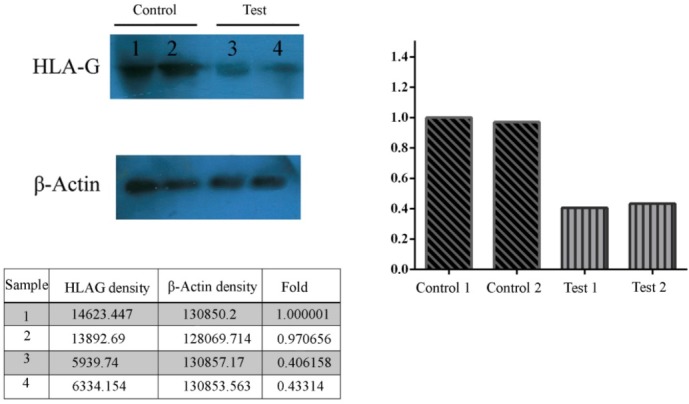
The results of immunoblotting. The evaluation of product bands from immunoblotting of HLA-G protein confirmed a decrease in expression of HLA-G in the placental tissue of subjects with RM compared with subjects who could give normal birth. The results showed that protein has been loaded equally among test and control groups.

Also, immunoblotting evaluation of HLA-G protein showed that the expression of HLA-G was significantly decreased in the placenta samples from test group compare to the control group. The results showed that, protein has been loaded equally among test and control groups. The results of immunoblotting of HLA-G and β-Actin proteins are shown in Figure 2.

**Figure 3:**
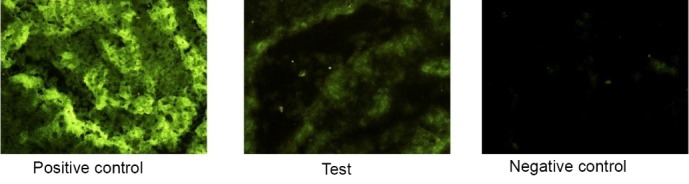
The results of fluorescent staining. Evaluation with fluorescence microscope showed a significant decrease in the expression of HLA-G protein in the placental tissue of subjects with RM compared with subjects who could give normal birth (positive control).

In addition, the fluorescent staining technique was used for analyze the expression of HLA-G protein in the studied tissues. The fluorescence microscope results showed a significant decrease of HLA-G protein expression in the placental tissue from test group compared with the control group. The evaluation of the negative control slides revealed that second FITC-conjugated antibodies had very low unspecific bound with the tissue samples, which could be ignored. The results of fluorescent staining are shown in Figure 3.

Although many studies have revealed the role of HLA-G in fetomaternal interface in the recent years, its association with successful pregnancy is still not completely understood. HLA-G is a member of nonclassical HLA class1b that plays an important role in inducing of tolerance to semi- allogeneic fetus in normal pregnancies.^25^ The membrane HLA-G is mostly pre-expressed on extra villous cytotrophoblast cells (ETVs) and sHLA-G expressed on placental trophoblasts of chorionic villous^26,27^ and have the capability of inhibiting NK cells, CD8+ and CD4+ T cells, B cells and antigen presenting cells such as macrophages and dendritic cells.^28^

In this study, HLA-G protein expression was measured in the subjects with RM in comparison with the healthy control. The results showed that HLA-G expression in RM subjects was decreased significantly. Recent studies revealed that HLA-G plays an important role in the pathogenesis of RM. Therefore, any deficiency in HLA-G expression is associated with recurrent miscarriage.^29^

Akhter et al showed that the expression of HLA-G in cultured EVTs cells of the subjects with RM was decreased significantly in comparison to the subjects with normal pregnancies.^30^ Also, similar results about decrease of HLA-G expression in EVTs cells have been suggested by various studies.^31^ However, the results of some other studies are not consistent with this study.^32^ Bhalla and colleagues evaluated the expression of HLA-G using immunohistochemistry staining in placental tissue of the subjects with RM samples compared with the healthy control group. The results showed that the rate of HLA-G expression is not significantly different between 2 groups. They declared that since the sample size in the similar studies were not adequate, so that their results are not consistent with the present study.^33^

Rabreau et al evaluated the rate of HLA-G expression in the patients with molar pregnancies and RM. They suggested that the rate of HLA-G expression was increased in molar pregnancy (a condition in which the trophoblastic cells are highly invasive), while in RM subjects, it was decreased in comparison with the normal pregnancies.^34^ It is assumed that the expression of HLA-G could affect the potency of trophoblast cells invasion. The lower expression of HLA-G has been reported in different studies.^35^

Most studies have evaluated the expression of HLA-G protein, but the results were controversial. In this study, as well as the evaluation of HLA-G protein expression rate, the gene expression of HLA-G gene was analyzed by using real-time PCR. The results indicated that, the HLA-G gene expression was decreased in the patients with RM. HLA-G alleles contains a 14 bp fragment in exon 8 that its deletion leads to significant decrease in expression of HLA-G mRNA and protein.^36,37^ Different studies showed that insertion/deletion polymorphism of this site is significantly associated with RM.^38,39^ These results can be indirectly in line with our findings in the present study. Although the association of this polymorphism with RM is controversial, other studies reported the association of different polymorphisms of HLA-G gene with recurrent miscarriages.^40,41^ Analyzing the results of different studies indicated that when the effect of single factors such as the rate of HLA-G expression, the level of sHLA-G, HLA-G polymorphisms and pro-inflammatory cytokines on RM are being assessed, the results are controversial. Supposedly, this inconsistency has not resulted in incorrect designing of studies, but probably from the insufficient samples size and incorrect way of selecting the RM subjects, since there are various classes of RM with different pathogenesis. In other words, in some studies patients with one or 2 abortion was considered as RM. Also, karyotyping of aborted fetus is an important issue because abortions that is resulted from genetic abnormality should not be considered as RM cases. Thus, in order to obtain better and more accurate results, it is suggested to conduct studies with greater sample size, more homogene samples and more clear definition of RM. Also, due to the heterogeneity and complicated histological structure of placenta, it is better to define the micro anatomical region of sampling comprehensively and more precise in the future studies.

Finally, we can be assumed that the synthetic sHLA-G analogs may be used for treatment of certain pregnancy-related disorders and facilitate reproduction. However, a fundamental understanding of the pathophysiology in these disorders is needed before starting to such expedience.

Also, in *in vitro* fertilization (IVF) treatments, the measurement of sHLA-G in the embryo culture medium can be used as a marker for improvement of successful assisted reproductive technology, by choosing the fertilized oocytes with highest potential, as sHLA-G positive culture medium correlates with pregnancy success.

## Conclusion

The present study showed that the decrement of HLA-G gene and protein expressions are associated with RM. But, since there are different controversial reports, it is suggested to conduct a more comprehensive similar study with greater sample size.

## Ethical Issues

The study was approved by the ethical committee of Tabriz university of medical science and all participants signed a written consent form before of the enrolment in this study (ethical code: 1530).

## Conflict of Interest

The authors have no conflicts of interest to declare.

## Acknowledgments

The authors would like to thank all of the Immunology Research Center, Tabriz University of Medical Sciences, for supporting the work.
